# Vaccination Coverage by Age 24 Months Among Children Born in 2021 and 2022 — National Immunization Survey-Child, United States, 2022–2024

**DOI:** 10.15585/mmwr.mm7511a2

**Published:** 2026-03-26

**Authors:** Holly A. Hill, David Yankey, Laurie D. Elam-Evans, Yi Mu, Michael Chen, Shannon Stokley, Georgina Peacock, James A. Singleton

**Affiliations:** 1 Immunization Services Division, National Center for Immunization and Respiratory Diseases, CDC.

**Summary**

**What is already known about this topic?**

When data were collected for this report in 2024, U.S. vaccination recommendations included routine vaccines and a monoclonal antibody to protect against 16 diseases among children by age 24 months.

**What is added by this report?**

Coverage with most vaccines was similar among children born during 2021–2022 and those born during 2019–2020. Decreases were observed for five vaccines. Coverage varied by Vaccines for Children (VFC) program eligibility, race and ethnicity, poverty status, urbanicity, and jurisdiction.

**What are the implications for public health practice?**

Efforts to improve and maintain high levels of vaccination coverage could help to reduce the morbidity and mortality associated with vaccine-preventable diseases. The Community Preventive Services Task Force recommends interventions such as the use of standing vaccination orders, immunization information systems, and vaccination programs in organized child care centers and in Special Supplemental Nutrition Program for Woman, Infants, and Children settings. Other factors demonstrated to be effective include strong provider recommendations, targeted messages from credible and trusted sources, and increased participation in the VFC program.

## Abstract

The National Immunization Survey-Child monitors coverage with recommended routine childhood vaccines. For data collected in survey year 2024, which include children born in 2021 and 2022, the household response rate (23.4%) and availability of adequate provider data for children with completed interviews (51.4%) were comparable to those from earlier survey years. For most vaccines, coverage by age 24 months was similar among children born in 2021 and 2022 and those born in 2019 and 2020. Declines in coverage of 1–2 percentage points were observed for the primary series of *Haemophilus influenzae* type b conjugate vaccine, the birth dose of hepatitis B vaccine, ≥4 doses of pneumococcal conjugate vaccine, and rotavirus vaccine. Coverage with ≥2 doses of influenza vaccine by age 24 months decreased from 61.0% among children born during 2019–2020 to 53.5% among those born during 2021–2022. Coverage was lower among Vaccines for Children (VFC) program–eligible children than among those who were not VFC-eligible and differed substantially by jurisdiction. Compared with non-Hispanic White children, coverage with many vaccines was lower among non-Hispanic Black or African American and Hispanic or Latino children; coverage was highest among non-Hispanic Asian children. Coverage was also lower among children living in poverty and those living in more rural areas. Maintaining high levels of vaccination and improving coverage among groups and in areas in which rates have declined could help protect children from vaccine-preventable morbidity and mortality. The Community Preventive Services Task Force recommends several interventions to increase vaccination, including standing orders for vaccination, immunization information systems, and vaccination programs in organized child care centers and in Special Supplemental Nutrition Program for Woman, Infants, and Children settings. Other factors demonstrated to be effective include strong provider recommendations, targeted messages from credible and trusted sources, and increased participation in the VFC program. 

## Introduction

As a public health strategy, immunization of young children has been critical to reducing morbidity and mortality due to vaccine-preventable diseases and has been found to be highly cost effective (*1*). During 2024, the most recent year of data collection available for this report, U.S. vaccination recommendations included receipt of routine vaccines and a monoclonal antibody to protect children against 16 potentially dangerous infections by age 24 months (*2*). For approximately 30 years, coverage with recommended childhood vaccines has been monitored by the National Immunization Survey-Child (NIS-Child).* Data from NIS-Child are used to estimate coverage at national, regional, state, and selected local area levels and for three U.S. territories (Guam, Puerto Rico, and the U.S. Virgin Islands).^†^ Data are stratified by the child’s year of birth, and vaccination status by age 24 months (or other milestone age) is assessed. This report describes trends in national coverage with recommended vaccines over time (excluding COVID-19 vaccine)^§^ and provides coverage estimates by Vaccines for Children (VFC) program eligibility,^¶^ race and ethnicity, poverty status, urbanicity, and jurisdiction of residence.

## Methods

### Data Collection

U.S. households that include children aged 19–35 months are identified through random-digit–dialing and invited to participate in NIS-Child. Household interviews with the adult most knowledgeable about the child’s vaccination history (usually a parent) are conducted via cellular telephone,** and consent is requested to contact the child’s vaccination providers and the state immunization registry. Once consent is obtained, a questionnaire is mailed to all of the child’s providers requesting detailed information about vaccines received by the child since birth. This information is synthesized into a comprehensive vaccination history for each child, which then serves as the basis for the coverage estimates included in this report.

Among households with eligible children identified in 2024 (the most recent survey year available), the household interview response rate^††^ was 23.4%, and adequate provider data^§§^ were available for 51.4% of children with completed interviews. NIS-Child uses a complex weighting process that includes adjustments for household nonresponse, provider nonresponse, and households without cellular telephones. Weights are calibrated to known population totals by age, sex, race and ethnicity, and geography to improve representation. Nonresponse adjustments are incorporated in the weighting to reduce potential bias. In addition, statistical modeling techniques such as imputation and variance estimation methods are used to handle missing data and account for the complex survey design (NIS-Child: A User's Guide). Children born during 2021–2022 were identified from data collected during survey years 2022–2024; data from 27,392 children were available for analysis. Application of survey weights to reflect the complex sample design of NIS-Child resulted in a weighted total sample size of 7,454,623.

### Data Analysis

Data from multiple survey years were combined and then stratified by year of birth to create birth cohorts for analysis. Kaplan-Meier techniques were used to estimate vaccination coverage by age 24 months for most vaccines. Exceptions include the birth dose of hepatitis B vaccine (HepB), which is considered received if administered during the first 3 days of life, and the rotavirus vaccine series, which is not meant to be given after age 8 months, 0 days. Because of a change in vaccination recommendations in 2020 and a long period of eligibility for catch-up vaccination, coverage with ≥2 doses of hepatitis A vaccine (HepA) was estimated by age 35 months (the maximum age within the scope of NIS-Child data collection) as well as by age 24 months.^¶¶^ Differences in coverage estimates were evaluated using z-tests at an α-level of 0.05. Estimated coverage nationally and by jurisdiction among children born during 2021 and 2022 was compared with that among children born during 2019 and 2020. For data stratified by sociodemographic characteristics, subgroup estimates were compared with those for a designated referent group. Analyses used weighted data and were performed using SAS software (version 9.4; SAS Institute) and SUDAAN software (version 11; RTI International). This activity was reviewed by CDC, deemed not research, and was conducted consistent with applicable federal law and CDC policy.***

## Results

### Recent Trends in National Vaccination Coverage by Birth Year

The largest difference in estimated vaccination coverage by age 24 months between children born in 2021 and 2022 and those born in 2019 and 2020 was a 7.4 percentage point decrease in coverage with ≥2 doses of influenza vaccine (Table 1). Smaller decreases were observed in coverage with the birth dose of HepB (1.8 percentage points), rotavirus vaccine (1.7), ≥4 doses of pneumococcal conjugate vaccine (PCV) (1.5), and the primary series of *Haemophilus influenzae* type b conjugate vaccine (Hib) (1.0). Nonsignificant decreases were observed for coverage with nearly all of the remaining vaccines. Coverage remained at or above 90.0% for ≥3 doses of poliovirus vaccine (92.1%), ≥3 doses of HepB (91.6%), ≥1 dose of measles, mumps, and rubella vaccine (MMR) (90.8%), and ≥1 dose of varicella vaccine (VAR) (90.0%). The lowest estimates of coverage were for ≥2 doses of HepA by age 24 months (46.8%) and ≥2 doses of influenza vaccine (53.5%). The percentage of children who received no vaccinations (1.2%) continued to meet the Healthy People 2030 target of ≤1.3%. Longer term trends (2011–2022) by single-year birth cohort reveal coverage consistently at or above 90.0% with ≥3 doses of poliovirus vaccine (range = 91.0%–93.8%), ≥1 dose of MMR (range = 89.8%–92.3%), ≥3 doses of HepB (range = 89.8%–92.6%), and ≥1 dose of VAR (range = 89.1%–91.2%) (Supplementary Figure). Although coverage with the birth dose of HepB increased by 10.6 percentage points during 2014–2019, coverage has steadily declined for the past three birth cohorts. Coverage with ≥2 doses of influenza vaccine declined significantly (12.0 percentage points) since 2019.

**TABLE 1:** Estimated vaccination coverage by age 24 months* among children born during 2019–2020 and 2021–2022 with selected vaccines and doses and percentage point differences in coverage between birth cohorts — National Immunization Survey-Child, United States, 2020–2024

Vaccine/Doses received	Birth years^†^ % (95% CI)		Percentage point difference (95% CI)
	2019–2020	2021–2022	2019–2020 to 2021–2022
DTaP^§^			
≥3 doses	93.3 (92.7 to 93.8)	92.7 (92.1 to 93.3)	−0.5 (−1.4 to 0.3)
≥4 doses	80.5 (79.5 to 81.4)	80.7 (79.7 to 81.6)	0.2 (−1.1 to 1.5)
Poliovirus (≥3 doses)	92.5 (91.9 to 93.0)	92.1 (91.5 to 92.8)	−0.3 (−1.2 to 0.5)
MMR (≥1 dose)^¶^	90.9 (90.2 to 91.5)	90.8 (90.1 to 91.5)	0 (–1.0 to 0.9)
Hib**			
Primary series	92.9 (92.3 to 93.4)	91.9 (91.2 to 92.5)	−1.0 (−1.8 to −0.1)^††^
Full series	78.8 (77.9 to 79.6)	77.6 (76.6 to 78.5)	−1.2 (−2.5 to 0.1)
HepB			
Birth dose^§§^	81.1 (80.3 to 81.8)	79.3 (78.3 to 80.2)	−1.8 (−3.0 to −0.6)^††^
≥3 doses	91.8 (91.2 to 92.4)	91.6 (91.0 to 92.3)	−0.1 (−1.0 to 0.8)
VAR (≥1 dose)^¶^	90.4 (89.7 to 91.1)	90.0 (89.3 to 90.7)	−0.4 (−1.4 to 0.6)
PCV			
≥3 doses	92.3 (91.7 to 92.9)	91.4 (90.8 to 92.1)	−0.9 (−1.7 to 0)
≥4 doses	82.0 (81.2 to 82.9)	80.5 (79.5 to 81.4)	−1.5 (−2.8 to −0.3)^††^
HepA^¶¶^			
≥1 dose	87.6 (86.9 to 88.3)	87.1 (86.3 to 87.8)	−0.5 (−1.6 to 0.5)
≥2 doses	47.5 (46.5 to 48.5)	46.8 (45.7 to 48.0)	−0.6 (−2.1 to 0.9)
≥2 doses (by age 35 mos)	79.3 (78.0 to 80.5)	78.7 (77.3 to 80.1)	−0.5 (−2.4 to 1.3)
Rotavirus (by age 8 mos)***	75.8 (74.9 to 76.7)	74.2 (73.1 to 75.2)	−1.7 (−3.0 to −0.3)^††^
Influenza (≥2 doses)^†††^	61.0 (60.0 to 61.9)	53.5 (52.4 to 54.6)	−7.4 (−8.9 to −5.9)^††^
Combined 7-vaccine series^§§§^	69.1 (68.1 to 70.1)	68.0 (67.0 to 69.1)	−1.0 (−2.5 to 0.4)
No vaccinations^¶¶¶^	1.2 (1.0 to 1.4)	1.2 (1.0 to 1.5)	0.1 (−0.2 to 0.4)

**Abbreviations:** DTaP = diphtheria, tetanus toxoids, and acellular pertussis vaccine; HepA = hepatitis A vaccine; HepB = hepatitis B vaccine; Hib = *Haemophilus influenzae* type b conjugate vaccine; MMR = measles, mumps, and rubella vaccine; PCV = pneumococcal conjugate vaccine; VAR = varicella vaccine.

* Includes vaccinations received by age 24 months (before the day the child turns 24 months), except for the HepB birth dose, rotavirus vaccination, and ≥2 HepA doses by age 35 months. For all vaccines except the HepB birth dose and rotavirus vaccination, the Kaplan-Meier method was used to estimate vaccination coverage to account for children whose vaccination history was ascertained before age 24 months (35 months for ≥2 HepA doses).

^†^ Data for the 2019 birth year are from survey years 2020, 2021, and 2022; data for the 2020 birth year are from survey years 2021, 2022, and 2023; data for the 2021 birth year are from survey years 2022, 2023, and 2024; and data for the 2022 birth year are considered preliminary and come from survey years 2023 and 2024 (data from survey year 2025 are not yet available).

^§^ Includes children who might have been vaccinated with diphtheria and tetanus toxoids vaccine or diphtheria, tetanus toxoids, and pertussis vaccine. Healthy People 2030 target for ≥4 doses of DTaP by age 2 years is 90.0%.

^¶^ Includes children who might have been vaccinated with measles, mumps, rubella, and varicella combination vaccine. Healthy People 2030 target for ≥1 dose of MMR by age 2 years is 90.8%.

** Hib primary series: receipt of ≥2 or ≥3 doses, depending on product type received; full series: primary series and booster dose, which includes receipt of ≥3 or ≥4 doses, depending on product type received.

^††^ Statistically significantly different (p<0.05) from zero (z-test).

^§§^ One dose of HepB administered from birth through age 3 days.

^¶¶^ In 2020, the HepA recommendation was revised to 2 doses at age 12–23 months, ≥6 months apart, with catch-up vaccination extending to age 18 years. Because of the prolonged eligibility period, coverage estimates for both age <24 months and age <35 months are provided.

*** Includes ≥2 doses of Rotarix monovalent rotavirus vaccine, or ≥3 doses of RotaTeq pentavalent rotavirus vaccine. (If any dose in the series is either RotaTeq or unknown, the recommendation defaults to the 3-dose series.) The maximum age for the final rotavirus dose is 8 months, 0 days.

^†††^ Doses must be ≥24 days apart (4 weeks with a 4-day grace period); doses could have been received during two influenza seasons.

^§§§^ The combined 7-vaccine series (4:3:1:3*:3:1:4) includes ≥4 doses of DTaP, ≥3 doses of poliovirus vaccine, ≥1 dose of measles-containing vaccine, the full series of Hib (≥3 or ≥4 doses, depending on product type), ≥3 doses of HepB, ≥1 dose of VAR, and ≥4 doses of PCV.

^¶¶¶^ Healthy People 2030 target for children who receive no recommended vaccines by age 2 years is ≤1.3%.

### Vaccination Coverage by Selected Sociodemographic Characteristics

Coverage with all vaccines was lower among children born in 2021 and 2022 who were eligible for VFC than among those who were not VFC-eligible (Table 2). Differences in coverage ranged from 2.5 percentage points (≥3 doses of HepB) to 22.4 percentage points (≥2 doses of influenza vaccine). By race and ethnicity, coverage with most vaccines was lower among non-Hispanic Black or African American children than among non-Hispanic White (White) children (Supplementary Table 1); exceptions include ≥1 dose of HepA, ≥2 doses of HepA (by age 35 months), the birth dose of HepB, ≥3 doses of HepB, and ≥1 dose of VAR. Compared with White children, coverage with ≥4 doses of diphtheria, tetanus toxoids, and acellular pertussis vaccine (DTaP) (5.5 percentage points), the full series of Hib (4.0), ≥4 doses of PCV (8.9), rotavirus (10.1), ≥2 doses of influenza vaccine (6.9), and the combined 7-vaccine series^†††^ (7.1) was lower among Hispanic or Latino children. Coverage with approximately one half of the vaccines assessed was higher among non-Hispanic Asian (Asian) children than among White children, with percentage point coverage differences ranging from 2.1 (≥3 doses of HepB) to 14.6 (≥2 influenza vaccine doses). Asian children were less likely than White children to have received no vaccinations (0.6% versus 1.4%). Coverage with all vaccines was lower among children living below the federal poverty level than among those living at or above the poverty level (Supplementary Table 2), with percentage point differences ranging from 3.5 (≥3 doses of HepB) to 18.1 (rotavirus). Compared with children living in a metropolitan statistical area (MSA)^§§§^ principal city (a measure of urbanicity), coverage with most vaccines was lower among children living in non-MSAs. Coverage among children living in an MSA nonprincipal city was lower (1.9 percentage points) only for ≥1 dose of HepA.

**TABLE 2:** Estimated vaccination coverage by age 24 months* among children born during 2021–2022,^†^ by selected vaccines and doses and Vaccines for Children eligibility status^§^ — National Immunization Survey-Child, United States, 2022–2024

Vaccine/Doses received	VFC eligibility status, % (95% CI)		Percentage point difference (95% CI)
	Not eligible (referent) n = 16,469	Eligible n = 10,923	
DTaP**^¶^**			
≥3 doses	94.8 (94.0 to 95.5)	90.7 (89.7 to 91.7)	−4.0 (−5.3 to −2.8)**
≥4 doses	85.5 (84.3 to 86.6)	76.0 (74.5 to 77.5)	−9.5 (−11.4 to −7.6)**
Poliovirus (≥3 doses)	94.1 (93.2 to 94.8)	90.3 (89.3 to 91.3)	−3.7 (−5.0 to −2.5)**
MMR (≥1 dose)^††^	92.6 (91.7 to 93.4)	89.2 (88.1 to 90.2)	−3.4 (−4.8 to −2.0)**
Hib^§§^			
Primary series	94.0 (93.2 to 94.7)	89.8 (88.8 to 90.8)	−4.2 (−5.5 to −2.9)**
Full series	81.9 (80.7 to 83.0)	73.4 (71.9 to 75.0)	−8.4 (−10.4 to −6.5)**
HepB			
Birth dose^¶¶^	81.8 (80.6 to 82.9)	76.9 (75.4 to 78.4)	−4.9 (−6.8 to −3.0)**
≥3 doses	92.9 (92.0 to 93.7)	90.4 (89.4 to 91.4)	−2.5 (−3.7 to −1.2)**
VAR (≥1 dose)^††^	91.8 (90.9 to 92.7)	88.3 (87.1 to 89.4)	−3.5 (−5.0 to −2.1)**
PCV			
≥3 doses	93.9 (93.1 to 94.6)	89.1 (88.1 to 90.1)	−4.8 (−6.1 to −3.5)**
≥4 doses	87.0 (85.9 to 90.3)	74.3 (72.7 to 75.8)	−12.7 (−12.6 to −10.8)**
HepA***			
≥1 dose	89.4 (89.5 to 91.7)	84.8 (83.5 to 86.0)	−4.6 (−6.3 to −3.1)**
≥2 doses	49.3 (47.9 to 50.7)	44.4 (42.6 to 46.3)	−4.9 (−7.2 to −2.6)**
≥2 doses (by age 35 mos)	82.9 (81.3 to 84.5)	74.8 (72.5 to 77.1)	−8.1 (−10.9 to −5.3)**
Rotavirus (by age 8 mos)^†††^	81.6 (80.5 to 82.8)	67.0 (65.3 to 68.6)	−14.7 (−16.7 to −12.6)**
Influenza (≥2 doses)^§§§^	64.9 (63.6 to 66.3)	42.5 (40.8 to 44.3)	−22.4 (−24.6 to 20.2)**
Combined 7-vaccine series^¶¶¶^	74.2 (72.9 to 75.4)	62.1 (60.4 to 63.8)	−12.0 (−14.1 to −9.9)**
No vaccinations	1.1 (0.8 to 1.6)	1.3 (1.0 to 1.6)	0.1 (−0.3 to 0.6)

**Abbreviations:** DTaP = diphtheria, tetanus toxoids, and acellular pertussis vaccine; HepA = hepatitis A vaccine; HepB = hepatitis B vaccine; Hib = *Haemophilus influenzae* type b conjugate vaccine; MMR = measles, mumps, and rubella vaccine; PCV = pneumococcal conjugate vaccine; VAR = varicella vaccine; VFC = Vaccines for Children.

* Includes vaccinations received before age 24 months, except for the HepB birth dose, rotavirus vaccination, and ≥2 HepA doses by age 35 months. For all vaccines except the HepB birth dose and rotavirus vaccination, the Kaplan-Meier method was used to estimate vaccination coverage to account for children whose vaccination history was ascertained before age 24 months (35 months for ≥2 HepA doses).

^†^ Data for the 2021 birth year are from survey years 2022, 2023, and 2024; data for the 2022 birth year are considered preliminary and come from survey years 2023 and 2024 (data from survey year 2025 are not yet available).

^§^ VFC eligibility was defined as meeting at least one of the following criteria: 1) American Indian or Alaska Native; 2) insured by Medicaid or Indian Health Service, or uninsured; or 3) ever received at least one vaccination at an Indian Health Service–operated center, Tribal health center, or urban Indian health care facility.

^¶^ Includes children who might have been vaccinated with diphtheria and tetanus toxoids vaccine or diphtheria and tetanus toxoids and pertussis vaccine.

** Statistically significant (p<0.05) difference compared with the referent group.

^††^ Includes children who might have been vaccinated with measles, mumps, rubella, and varicella combination vaccine.

^§§^ Hib primary series: receipt of ≥2 or ≥3 doses, depending on product type received; full series: primary series and booster dose, which includes receipt of ≥3 or ≥4 doses, depending on product type received.

^¶¶^ One dose HepB administered from birth through age 3 days.

*** Before 2020, a first dose of HepA was recommended at age 12–23 months, with the second dose 6–18 months after the first, depending upon the product type received. In 2020, the recommendation was revised to 2 doses at age 12–23 months, ≥6 months apart. Because children in this analysis were vaccinated under both recommendations, coverage estimates for both age <24 months and age <35 months are provided.

^†††^ Includes ≥2 doses of Rotarix monovalent rotavirus vaccine, or ≥3 doses of RotaTeq pentavalent rotavirus vaccine. (If any dose in the series is either RotaTeq or unknown, the recommendation defaults to the 3-dose series.) The maximum age for the final rotavirus dose is 8 months, 0 days.

^§§§^ Doses must be ≥24 days apart (4 weeks with a 4-day grace period); doses could have been received during two influenza seasons.

^¶¶¶^ The combined 7-vaccine series (4:3:1:3*:3:1:4) includes ≥4 doses of DTaP, ≥3 doses of poliovirus vaccine, ≥1 dose of measles-containing vaccine, the full series of Hib (≥3 or ≥4 doses, depending on product type), ≥3 doses of HepB, ≥1 dose of VAR, and ≥4 doses of PCV.

### Vaccination Coverage by Jurisdiction

Variation in coverage with selected vaccines was also observed by jurisdiction (Table 3), especially for ≥2 doses of influenza vaccine, which ranged from 25.2% (Mississippi) to 78.3% (Massachusetts). Coverage with ≥2 doses of influenza vaccine among children born during 2021–2022 decreased compared with coverage among children born during 2019–2020 in 30 (54.6%) of 56 states and local areas.

**TABLE 3:** Estimated vaccination coverage by age 24 months* among children born during 2021–2022,^†^ by selected vaccines and doses, overall and by area^§^ — National Immunization Survey-Child, United States, 2022–2024

Area	No.	Vaccine coverage, % (95% CI)							
		MMR^¶^ (≥1 dose)	Poliovirus (≥3 doses)	DTaP** (≥4 doses)	HepB^††^ (birth dose)	HepA (≥2 doses by age 35 mos)	Rotavirus^§§^	Influenza^¶¶^ (≥2 doses)	Combined 7-vaccine series***
**United States**	**27,392**	**90.8 (90.1–91.5)**	**92.1 (91.5–92.8)**	**80.7 (79.7–81.6)**	**79.3 (78.3–80.2)^†††^**	**78.7 (77.3–80.1)**	**74.2 (73.1–75.2)^†††^**	**53.5 (52.4–54.6)^†††^**	**68.0 (67.0–69.1)**
**HHS Region 1, total**	**2,580**	**95.1 (93.5–96.4)**	**95.7 (94.1–97.0)**	**88.9 (86.5–91.1)**	**87.3 (85.1–89.1)**	**89.2 (85.5–92.3)**	**83.1 (80.2–85.6)**	**75.0 (72.1–77.9)**	**80.4 (77.6–83.0)**
Connecticut	390	95.6 (92.5–97.7)	95.5 (92.0–97.8)	89.8 (85.2–93.4)	88.9 (84.5–92.2)	92.8 (86.5–96.9)	83.9 (77.4–88.8)	72.9 (65.9–79.6)^†††^	79.9 (74.2–85.0)
Maine	361	93.4 (89.6–96.1)	96.3 (93.5–98.1)	82.0 (75.4–87.8)	81.6 (75.3–86.5)	85.1 (76.8–91.6)	79.8 (74.5–84.2)	66.9 (60.3–73.4)	68.7 (62.0–75.2)
Massachusetts	452	94.9 (91.8–97.1)	95.2 (92.1–97.3)	89.4 (85.0–93.0)	88.7 (84.8–91.7)	88.8 (81.3–94.2)	83.1 (78.0–87.3)	78.3 (73.5–82.6)	82.5 (77.7–86.8)
New Hampshire	354	94.7 (91.7–96.9)	96.0 (93.4–97.8)	90.0 (86.2–93.1)^§§§^	82.8 (76.8–87.6)	89.7 (83.6–94.3)^§§§^	83.4 (77.9–87.8)	73.0 (67.3–78.4)	80.9 (75.9–85.5)
Rhode Island	555	95.4 (92.7–97.4)	96.7 (94.1–98.4)	90.5 (86.7–93.6)	86.5 (82.9–89.5)^§§§^	94.3 (82.3–99.1)	83.1 (78.9–86.5)^†††^	74.0 (69.2–78.6)^†††^	80.2 (75.9–84.3)
Vermont	468	97.4 (94.7–98.9)	97.5 (95.0–99.0)	87.1 (81.9–91.4)	82.5 (78.0–86.3)	81.1 (71.9–88.7)	83.5 (78.9–87.2)	72.4 (66.8–77.7)^†††^	80.6 (75.4–85.2)
**HHS Region 2, total**	**1,463**	**89.3 (86.6–91.6)^†††^**	**90.8 (88.4–92.9)**	**79.4 (76.0–82.7)**	**75.2 (71.7–78.4)^†††^**	**69.6 (64.5–74.6)^†††^**	**71.8 (68.4–75.0)**	**58.9 (55.3–62.5)^†††^**	**63.9 (60.3–67.5)**
New Jersey	463	87.7 (82.8–91.8)^†††^	90.6 (86.5–93.8)	77.9 (71.0–84.1)	79.7 (72.7–85.2)	70.7 (61.6–79.2)	72.7 (66.6–78.1)	59.2 (52.6–65.9)^†††^	61.3 (54.6–68.1)
New York	1,000	90.1 (86.8–92.9)	90.9 (87.8–93.5)	80.2 (76.3–83.8)	72.8 (68.7–76.6)^†††^	69.0 (62.7–75.0)	71.3 (67.1–75.1)^†††^	58.7 (54.5–62.9)^†††^	65.3 (61.1–69.4)
New York City	552	89.3 (85.3–92.6)	91.1 (87.5–94.0)	80.3 (75.4–84.7)	74.4 (69.2–78.9)	71.6 (63.9–79.0)	73.2 (68.1–77.8)	61.6 (56.3–67.0)	67.6 (62.5–72.7)
New York, excluding New York City	448	90.8 (85.4–94.8)	90.8 (85.7–94.6)	80.1 (74.2–85.5)	71.6 (65.2–77.2)	66.2 (56.9–75.3)	69.7 (63.3–75.4)^†††^	56.3 (50.1–62.6)	63.4 (57.2–69.6)
**HHS Region 3, total**	**3,790**	**91.6 (90.0–92.9)**	**92.9 (91.5–94.2)**	**82.3 (80.2–84.3)**	**81.2 (78.9–83.3)**	**82.1 (78.7–85.3)**	**80.6 (78.5–82.5)**	**61.2 (58.6–63.8)^†††^**	**71.5 (69.1–73.8)**
Delaware	294	91.4 (86.9–94.9)	94.5 (91.0–97.0)	84.2 (79.1–88.7)	83.7 (77.6–88.4)	81.7 (74.8–87.6)^†††^	76.3 (68.9–82.4)	55.9 (48.6–63.5)^†††^	70.3 (63.4–76.9)
District of Columbia	526	87.4 (80.7–92.6)	86.3 (79.0–92.0)	70.3 (62.8–77.5)	80.1 (73.7–85.2)	73.1 (62.9–82.5)	69.4 (61.4–76.4)	61.1 (53.7–68.7)	62.5 (55.0–70.1)
Maryland	793	95.2 (92.5–97.2)	95.1 (92.2–97.2)	84.1 (79.4–88.2)	81.1 (76.7–84.8)	79.2 (72.9–84.9)	83.2 (79.2–86.6)	62.8 (57.8–67.9)	72.8 (67.8–77.5)
Pennsylvania	1,027	89.7 (86.8–92.2)	92.0 (89.4–94.2)	84.0 (80.6–87.2)	84.1 (80.4–87.2)	84.5 (77.1–90.6)	79.9 (76.2–83.2)	62.2 (57.8–66.7)^†††^	72.2 (68.0–76.3)
Philadelphia	480	91.7 (87.6–94.9)	91.4 (87.7–94.3)	82.4 (76.8–87.3)	81.3 (74.9–86.3)	80.7 (71.7–88.4)	75.3 (69.3–80.5)	66.4 (60.3–72.4)	72.7 (66.6–78.5)
Pennsylvania, excluding Philadelphia	547	89.3 (86.0–92.1)	92.1 (89.1–94.6)	84.4 (80.4–87.9)	84.5 (80.3–88.0)	85.2 (76.5–92.0)	80.7 (76.5–84.4)	61.5 (56.4–66.6)^†††^	72.1 (67.3–76.8)
Virginia	691	91.2 (88.1–93.8)	92.4 (89.4–94.8)	79.1 (74.6–83.3)	77.3 (72.0–81.8)	81.4 (75.6–86.6)	81.0 (76.8–84.6)	61.7 (56.5–66.9)^†††^	70.4 (65.7–74.9)
West Virginia	459	94.5 (91.8–96.5)^§§§^	95.5 (93.1–97.2)^§§§^	83.9 (79.5–87.8)^§§§^	80.7 (76.1–84.6)	88.4 (80.0–94.4)^§§§^	79.4 (74.9–83.2)^§§§^	48.0 (42.6–53.7)	71.4 (66.3–76.2)^§§§^
**HHS Region 4, total**	**4,226**	**91.0 (89.7–92.2)**	**92.8 (91.6–93.9)**	**80.6 (78.6–82.5)**	**76.3 (74.2–78.3)^†††^**	**77.1 (74.0–80.0)**	**73.4 (71.3–75.5)**	**42.2 (39.9–44.6)^†††^**	**66.9 (64.7–69.2)**
Alabama	454	89.1 (84.5–92.8)^†††^	88.3 (83.5–92.2)^†††^	80.0 (74.4–85.1)	78.2 (72.6–83.0)	75.8 (68.9–82.2)	67.6 (60.9–73.7)^†††^	32.2 (26.9–38.2)	67.2 (60.9–73.4)
Florida	636	92.0 (89.1–94.4)	93.6 (90.7–95.9)	81.9 (77.5–86.0)	74.7 (69.9–79.1)	75.4 (68.1–82.1)	74.1 (69.2–78.5)	39.8 (34.6–45.5)	72.5 (67.7–77.2)
Georgia	569	87.4 (82.9–91.2)	91.9 (88.3–94.8)	76.2 (70.5–81.5)	77.9 (72.7–82.3)	80.1 (73.1–86.3)	74.9 (69.6–79.6)	41.5 (36.1–47.5)	58.7 (52.7–64.8)^†††^
Kentucky	519	90.3 (86.3–93.5)	91.5 (87.3–94.8)	79.5 (74.2–84.4)	78.4 (73.2–82.8)	81.7 (71.8–89.7)	74.5 (69.3–79.1)	43.9 (38.7–49.4)	66.3 (60.8–71.6)
Mississippi	428	88.4 (81.5–93.6)	88.3 (81.8–93.3)	77.2 (69.9–83.8)	79.2 (72.4–84.6)	56.0 (46.5–65.9)	61.2 (53.8–68.2)	25.2 (20.1–31.4)^†††^	64.4 (57.0–71.8)
North Carolina	642	94.4 (92.3–96.1)	95.1 (92.5–97.0)	82.0 (77.8–85.9)	76.6 (70.9–81.4)^†††^	76.6 (70.2–82.5)	76.6 (70.8–81.4)	50.2 (45.0–55.7)^†††^	67.1 (61.7–72.4)^†††^
South Carolina	460	90.8 (87.0–93.8)	92.8 (89.7–95.2)	84.0 (79.3–88.2)	79.5 (71.8–85.4)	79.4 (71.2–86.6)	79.8 (74.4–84.4)	40.0 (33.5–47.2)	68.0 (62.2–73.7)
Tennessee	518	91.6 (88.5–94.2)	94.6 (92.2–96.4)	81.1 (76.1–85.6)	72.0 (66.4–76.9)	83.1 (75.5–89.5)	68.5 (62.4–74.0)	52.4 (46.7–58.4)	64.0 (58.4–69.7)
**HHS Region 5, total**	**3,532**	**92.0 (90.6–93.2)**	**92.5 (91.2–93.7)**	**83.7 (81.9–85.5)**	**82.7 (80.8–84.4)**	**80.7 (77.9–83.3)**	**78.0 (75.9–79.9)**	**58.4 (56.1–60.7)^†††^**	**72.7 (70.6–74.7)^§§§^**
Illinois	883	93.0 (90.2–95.1)^§§§^	94.4 (91.9–96.3)	81.1 (77.3–84.7)	80.0 (76.3–83.2)	80.6 (74.9–85.6)	76.0 (72.0–79.6)	61.1 (56.8–65.4)	68.7 (64.5–72.7)
Chicago	247	92.9 (87.3–96.6)	93.9 (89.0–97.1)	81.6 (73.9–88.2)	78.8 (69.8–85.7)	84.9 (76.0–91.8)	78.3 (69.5–85.1)	63.8 (55.3–72.3)	73.0 (64.9–80.5)
Illinois, excluding Chicago	636	93.0 (89.8–95.5)	94.5 (91.5–96.7)	81.0 (76.5–85.1)	80.3 (76.3–83.8)	79.6 (72.8–85.6)	75.4 (70.7–79.5)	60.3 (55.3–65.3)	67.5 (62.7–72.2)
Indiana	351	90.8 (86.5–94.1)	90.1 (85.5–93.7)	82.0 (76.7–86.7)	81.0 (75.0–85.7)	79.8 (72.5–86.2)	78.9 (73.1–83.7)	51.5 (44.9–58.5)	74.3 (68.1–80.0)
Michigan	718	92.4 (88.6–95.3)	92.3 (88.9–95.0)	82.9 (77.9–87.4)	80.7 (75.0–85.3)	83.7 (76.0–90.0)	74.7 (68.8–79.7)	57.7 (52.1–63.4)^†††^	73.6 (68.5–78.4)
Minnesota	444	91.3 (87.4–94.4)	93.4 (90.2–95.9)	87.5 (83.1–91.2)	86.6 (82.5–89.9)^§§§^	78.6 (71.2–85.1)	81.4 (76.4–85.5)	68.4 (62.6–74.1)	72.0 (66.5–77.3)
Ohio	655	93.8 (91.1–95.9)	92.5 (89.4–94.9)	85.4 (81.3–89.1)	84.4 (80.3–87.7)	77.5 (71.1–83.3)	79.1 (74.6–83.1)	52.9 (47.7–58.3)^†††^	73.2 (68.4–77.7)
Wisconsin	481	87.5 (82.2–91.9)	91.4 (87.9–94.1)	85.1 (80.8–88.9)	86.2 (81.0–90.1)	83.9 (76.6–89.9)	80.3 (73.3–85.8)	64.0 (57.9–70.1)	77.0 (71.4–82.2)^§§§^
**HHS Region 6, total**	**3,347**	**91.0 (89.2–92.7)**	**91.3 (89.5–93.0)**	**79.5 (76.8–82.1)**	**79.3 (76.5–81.8)**	**78.9 (74.2–83.3)**	**69.7 (66.5–72.7)^†††^**	**44.9 (41.7–48.3)^†††^**	**64.9 (61.8–68.0)**
Arkansas	456	89.1 (84.6–92.7)	91.2 (87.1–94.5)	77.5 (71.9–82.7)	88.0 (83.7–91.4)^§§§^	76.6 (69.5–83.1)	74.6 (68.7–79.6)	36.6 (31.1–42.8)^†††^	65.5 (59.5–71.5)
Louisiana	642	90.8 (86.5–94.2)	94.1 (90.4–96.7)	80.3 (74.6–85.4)	78.0 (71.9–83.1)	75.9 (68.6–82.7)	75.9 (70.2–80.8)	34.7 (29.5–40.6)^†††^	68.2 (62.1–74.3)
New Mexico	538	90.7 (87.4–93.4)	92.1 (89.2–94.5)	79.3 (74.3–83.9)	78.7 (73.4–83.1)	73.7 (66.6–80.3)^†††^	78.7 (73.9–82.9)	51.1 (45.4–57.1)^†††^	68.7 (63.3–73.9)^†††^
Oklahoma	356	90.5 (86.1–94.0)	88.5 (83.1–92.8)	74.0 (67.7–80.0)	77.2 (71.2–82.3)	87.4 (78.8–93.7)^§§§^	66.4 (59.5–72.7)	39.3 (33.3–46.0)^†††^	61.7 (55.0–68.4)
Texas	1,355	91.3 (88.8–93.4)	91.3 (88.8–93.4)	80.3 (76.7–83.7)	79.0 (75.2–82.3)	78.5 (72.2–84.3)	68.3 (64.1–72.3)^†††^	47.5 (43.1–52.0)	64.6 (60.4–68.8)
Bexar County	410	93.2 (90.2–95.6)^§§§^	90.9 (86.1–94.5)	81.6 (76.1–86.5)^§§§^	75.4 (69.0–80.9)	80.2 (72.2–87.1)	70.4 (63.5–76.5)	52.0 (45.6–58.8)	65.2 (58.7–71.6)
Houston	343	88.3 (83.3–92.4)	89.1 (84.1–93.1)	75.1 (68.6–81.2)	78.7 (73.0–83.6)	—^¶¶¶^	69.4 (62.5–75.4)	45.8 (39.5–52.7)^†††^	61.0 (54.2–67.9)
Texas, excluding Bexar County and Houston	602	91.6 (88.6–94.0)	91.6 (88.6–94.1)	81.0 (76.6–85.0)	79.3 (74.7–83.2)	78.4 (70.6–85.2)	68.0 (62.9–72.8)^†††^	47.3 (42.1–52.9)	65.0 (60.0–70.0)
**HHS Region 7, total**	**2,049**	**92.9 (91.4–94.3)^§§§^**	**93.3 (91.8–94.6)**	**81.5 (79.0–84.0)**	**82.5 (80.3–84.5)**	**80.0 (76.0–83.6)**	**78.3 (75.7–80.6)**	**54.0 (51.1–57.0)^†††^**	**69.6 (66.8–72.4)**
Iowa	454	95.0 (92.1–97.1)	96.7 (94.5–98.2)	86.0 (81.4–90.0)	83.6 (79.0–87.4)	73.5 (65.3–81.1)	76.7 (71.3–81.3)	48.2 (42.5–54.2)	76.8 (71.6–81.6)
Kansas	610	89.9 (86.6–92.6)	89.2 (85.8–92.1)	78.0 (73.2–82.5)	78.2 (73.7–82.1)	81.4 (75.2–86.8)	70.8 (65.7–75.3)^†††^	45.3 (40.1–50.7)^†††^	65.8 (60.6–71.0)
Missouri	581	93.0 (90.1–95.3)^§§§^	93.3 (90.5–95.5)	80.1 (75.2–84.6)	83.8 (79.8–87.1)	82.8 (75.7–88.8)	81.7 (77.0–85.6)^§§§^	57.5 (52.2–62.9)	69.8 (64.6–74.8)
Nebraska	404	94.0 (90.6–96.6)	94.1 (91.2–96.3)	83.9 (79.1–88.1)	83.3 (78.2–87.4)	79.5 (72.8–85.5)	82.2 (77.0–86.4)	65.8 (60.0–71.5)^†††^	63.7 (57.9–69.5)
**HHS Region 8, total**	**2,604**	**90.0 (88.1–91.7)**	**92.4 (90.6–93.9)**	**80.1 (77.4–82.6)**	**82.4 (80.1–84.5)**	**82.8 (79.1–86.1)**	**76.7 (74.1–79.2)**	**58.4 (55.3–61.5)^†††^**	**69.5 (66.6–72.4)**
Colorado	479	90.2 (86.9–93.0)	92.2 (88.8–94.8)	79.7 (74.6–84.3)	82.0 (77.8–85.6)	82.9 (74.9–89.5)	78.6 (74.0–82.5)	64.8 (59.4–70.2)^†††^	69.0 (63.7–74.2)
Montana	373	87.0 (81.9–91.3)	91.7 (88.1–94.6)	74.7 (68.8–80.3)	79.3 (74.6–83.4)	71.5 (63.6–78.9)	68.7 (62.5–74.3)	47.6 (41.8–53.7)^†††^	67.1 (61.0–73.0)
North Dakota	384	88.3 (83.1–92.5)	90.7 (86.6–93.9)	79.6 (73.4–85.2)	85.1 (79.9–89.0)	75.0 (66.9–82.4)^†††^	77.6 (72.0–82.5)	61.6 (55.1–68.2)^†††^	69.1 (62.7–75.3)
South Dakota	424	92.2 (88.7–95.0)	93.0 (89.6–95.5)	75.7 (69.3–81.6)	85.7 (78.5–90.7)	81.7 (71.5–89.9)	75.0 (68.3–80.7)	57.5 (50.7–64.6)^†††^	64.2 (57.4–71.0)
Utah	481	90.9 (87.3–93.8)	93.5 (90.1–96.1)	83.4 (78.7–87.6)	83.2 (78.6–86.9)	89.8 (84.3–94.1)^§§§^	77.1 (71.6–81.8)	53.3 (47.6–59.3)^†††^	73.0 (67.5–78.2)
Wyoming	463	87.2 (82.8–90.9)	89.4 (85.3–92.8)	75.5 (70.0–80.7)	76.9 (71.3–81.6)	63.6 (56.9–70.2)	73.3 (67.6–78.3)	48.4 (42.6–54.5)^†††^	65.1 (59.4–70.8)
**HHS Region 9, total**	**1,958**	**87.6 (84.3–90.6)**	**90.5 (87.6–93.0)**	**75.8 (71.7–79.7)**	**77.1 (72.9–80.8)**	**78.2 (72.7–83.3)**	**68.6 (64.1–72.8)**	**56.0 (51.7–60.4)^†††^**	**63.7 (59.5–68.0)**
Arizona	438	87.8 (82.9–91.9)	94.4 (91.2–96.8)^§§§^	78.4 (72.0–84.2)	83.5 (77.4–88.2)	77.9 (69.9–85.0)	76.7 (70.3–82.0)	51.8 (45.3–58.7)	69.0 (62.6–75.2)
California	590	87.3 (83.0–90.9)	89.5 (85.7–92.6)	74.7 (69.6–79.6)	75.4 (70.0–80.1)	77.9 (70.9–84.2)	66.4 (60.6–71.7)	57.8 (52.3–63.4)^†††^	62.1 (56.7–67.6)
Hawaii	378	88.5 (84.0–92.3)	91.9 (88.6–94.6)	82.4 (76.5–87.5)	86.2 (81.3–90.0)	78.9 (69.2–87.2)	76.1 (70.4–80.9)	60.8 (54.2–67.4)	70.8 (64.4–76.8)
Nevada	552	92.1 (88.9–94.7)^§§§^	93.9 (91.4–95.9)	78.6 (73.2–83.5)	78.6 (73.0–83.2)	84.2 (75.8–90.9)^§§§^	73.2 (67.9–77.8)	41.9 (36.2–48.0)^†††^	67.3 (61.5–72.9)
**HHS Region 10, total**	**1,843**	**92.4 (90.7–93.9)^§§§^**	**92.4 (90.6–93.9)**	**81.8 (79.2–84.3)**	**80.1 (77.3–82.6)**	**79.1 (75.1–83.0)**	**75.1 (72.2–77.7)**	**60.9 (57.8–64.1)^†††^**	**69.0 (65.9–71.9)**
Alaska	405	83.0 (75.5–89.2)	83.7 (76.2–89.9)	66.4 (58.6–74.2)	75.9 (69.0–81.7)	69.3 (60.0–78.2)	66.6 (58.6–73.8)	43.3 (36.6–50.6)^†††^	53.0 (45.5–60.9)
Idaho	426	90.6 (87.0–93.6)	89.4 (85.0–93.0)	79.0 (73.9–83.7)	78.3 (73.2–82.6)	80.4 (72.2–87.4)	74.7 (69.0–79.6)	49.9 (44.2–55.9)^†††^	72.1 (66.7–77.2)
Oregon	407	92.7 (89.7–95.1)	92.5 (89.3–95.0)	80.9 (76.1–85.4)	82.9 (78.3–86.8)	77.8 (71.2–83.7)	73.7 (68.2–78.5)	61.4 (55.9–67.0)	65.4 (59.9–70.9)
Washington	605	93.5 (90.7–95.7)	93.9 (91.3–96.0)	84.4 (80.4–88.0)	79.6 (75.0–83.6)	80.4 (73.6–86.4)	76.7 (72.3–80.6)	65.5 (60.6–70.3)^†††^	71.5 (66.8–76.0)
**Range of column values, states**	NA	83.0–97.4	83.7−97.5	66.4–90.5	72.0–88.9	56.0–94.3	61.1–83.9	25.2–78.3	53.0–82.5
Territory****									
Guam	117	—^¶¶¶^	—^¶¶¶^	—^¶¶¶^	84.7 (72.8–92.0)	—^¶¶¶^	—^¶¶¶^	—^¶¶¶^	—^¶¶¶^
Puerto Rico	523	77.5 (72.2–82.5)^§§§^	80.1 (74.9–84.9)	64.9 (58.7–71.0)^§§§^	73.7 (67.8–78.8)	—^¶¶¶^	59.7 (53.1–66.0)	16.8 (12.3–22.9)	52.4 (45.7–59.3)^§§§^

**Abbreviations:** DTaP = diphtheria, tetanus toxoids, and acellular pertussis vaccine; HepA = hepatitis A vaccine; HepB = hepatitis B vaccine; HHS = U.S. Department of Health and Human Services; Hib = *Haemophilus influenzae* type b conjugate vaccine; MMR = measles, mumps, and rubella vaccine; NA = not applicable; PCV = pneumococcal conjugate vaccine.

* Includes vaccinations received by age 24 months (before the day the child turns 24 months), except for the HepB birth dose, rotavirus vaccination, and ≥2 HepA doses by age 35 months. For all vaccines except the HepB birth dose and rotavirus vaccination, the Kaplan-Meier method was used to estimate vaccination coverage to account for children whose vaccination history was ascertained before age 24 months (35 months for ≥2 HepA doses).

^†^ Data for the 2021 birth year are from survey years 2022, 2023, and 2024; data for the 2022 birth year are considered preliminary and are from survey years 2023 and 2024 (2025 data are not yet available).

**^§^** HHS Regional Offices | HHS.gov

^¶^ Includes children who might have been vaccinated with measles, mumps, rubella, and varicella combination vaccine.

** Includes children who might have been vaccinated with diphtheria and tetanus toxoids vaccine or diphtheria, tetanus toxoids, and pertussis vaccine.

^††^ One dose HepB administered from birth through age 3 days.

^§§^ Includes ≥2 doses of Rotarix monovalent rotavirus vaccine, or ≥3 doses of RotaTeq pentavalent rotavirus vaccine. (If any dose is RotaTeq or unknown, the recommendation defaults to the 3-dose series.) The maximum age for the final rotavirus dose is 8 months, 0 days.

^¶¶^ Doses must be ≥24 days apart (4 weeks with a 4-day grace period); doses could have been received during two influenza seasons.

*** The combined 7-vaccine series (4:3:1:3*:3:1:4) includes ≥4 doses of DTaP, ≥3 doses of poliovirus vaccine, ≥1 dose of measles-containing vaccine, the full series of Hib (≥3 or ≥4 doses, depending on product type), ≥3 doses of HepB, ≥1 dose of varicella vaccine, and ≥4 doses of PCV. Coverage estimates for vaccines not included in this table are available online. Vaccination Coverage among Young Children (0 – 35 Months) | ChildVaxView | CDC

^†††^ Statistically significant decrease in estimated coverage compared with children born during 2019–2020 (p<0.05).

^§§§^ Statistically significant increase in estimated coverage compared with children born in 2019–2020 (p<0.05).

^¶¶¶^ Estimate not available because the unweighted sample size for the denominator was <30, or 95% CI half-width / estimate >0.588, or 95% CI half-width was ≥10.

**** Sample size was too small to calculate reliable coverage estimates for the U.S. Virgin Islands, because data were not collected there in 2022 and 2024.

## Discussion

For most vaccines monitored by NIS-Child, estimated coverage by age 24 months for children born in 2021 and 2022 was similar to coverage among those born in 2019 and 2020. Healthy People 2030 objectives were met for ≥1 dose of MMR (target ≥90.8% versus 90.8% achieved) and receipt of no vaccinations (target ≤1.3% versus 1.2% achieved) but not for ≥4 doses of DTaP (target ≥90.0% versus 80.7% achieved). Although coverage with most vaccines has been maintained, the decline in coverage with the HepB birth dose for the past three birth cohorts is a notable trend. The birth dose, recommended within 24 hours of birth, according to the immunization schedule that was in place for all children in this study, serves as a universal safeguard against early hepatitis B virus (HBV) transmission. For infants born to mothers who are hepatitis B surface antigen (HBsAg)–positive, the birth dose, administered together with hepatitis B immune globulin, provides the critical first line of protection against perinatal HBV infection. Without this protection, approximately 90% of U.S. infants born to women who are HBsAg-positive will develop chronic infection with HBV, and approximately 25% of them will eventually die from chronic liver disease (Clinical Overview of Perinatal Hepatitis B | CDC). The HepB birth dose is also an important safety net protecting against HBV infection for infants born to the 12%–16% of pregnant women in the United States who, despite having health insurance and receiving prenatal care, are not tested for HBsAg during their pregnancy (*3*). Infection can also be transmitted through contact with blood or fluids from HBV-infected family or community members before infants have the opportunity to complete the 3-dose vaccination series.

A large decline in coverage with ≥2 doses of influenza vaccine resulted in its lowest level in more than a decade (51.8%). Vaccination against influenza decreased among children during the COVID-19 pandemic and has not yet recovered to prepandemic levels (*4*). A recent study among children aged 6 months–17 years reported that 30.9% of children had a parent hesitant about influenza vaccination, with higher prevalences of hesitancy among parents of younger children (*5*). Common reasons reported by parents for not obtaining an influenza vaccination for their children include a lack of belief that their child would get very sick from influenza (48.2%), concern about vaccine safety and side effects (43.3%), and a perception that the vaccine was not highly effective (37.0%) (*4*). The lack of concern over severity of influenza is relevant given that during the 2024–25 U.S. influenza season, the cumulative influenza-associated hospitalization rate was the highest since 2010–11 (*6*), and 280 pediatric deaths caused by influenza were reported, exceeding the highest number reported during a nonpandemic season since pediatric influenza deaths became reportable in 2004; 89% of those deaths occurred in children who were not fully vaccinated against influenza (*7*).

Differences in vaccination coverage by sociodemographic characteristics such as race and ethnicity, poverty status, MSA status, health insurance status, and eligibility for the VFC program persist, all of which have been documented in previous studies (*8*–*10*). VFC is designed to ensure that all children have access to vaccines, regardless of their family’s ability to pay. Growing a robust network of VFC-enrolled providers and ensuring the program reaches eligible children are essential to guaranteeing that all children have access to vaccination services.

### Limitations

The findings in this report are subject to at least three limitations. First, the household response rates (22%–27% during survey years 2020–2024) and the availability of adequate provider data for only approximately one half of those with completed household interviews during these survey years could lead to selection bias that was not completely eliminated by the use of survey weighting adjustments. Without adequate information about study nonparticipants, the direction of such bias is unknown. Second, both the sampling procedure and the collection of data by household interview rely on respondents having cellular telephones; omission of households without cellular telephones could also be a source of selection bias, although the effect would likely be small: according to a 2025 Pew Research Center survey, 98% of adults in the United States own a cellular telephone. Finally, vaccination histories could be incomplete if not all providers were identified by interview respondents or if some providers did not respond to requests for vaccination information. Previous assessments of total survey error in NIS-Child have indicated that vaccination coverage is underestimated by up to 9 percentage points for certain vaccines. The 2024 total survey error estimates were similar to those from previous years for the vaccines assessed (NORC at the University of Chicago, CDC, unpublished data, 2025).

### Implications for Public Health Practice

 Vaccines have substantially reduced severe illness, hospitalization, and death and have saved approximately $2.7 trillion in societal costs (*1*). Although national vaccination coverage remained stable for most vaccines, lower coverage among certain population subgroups and in some jurisdictions is creating an increased risk for outbreaks of vaccine-preventable diseases. During 2025, a total of 2,144 confirmed measles cases were reported in the United States, the largest number of annual cases since measles was declared eliminated in 2000. Among these cases, 93% occurred in persons who were not vaccinated against measles or whose vaccination status was unknown. The preliminary number of reported pertussis cases in 2024 was higher than that reported in 2019, before the COVID-19 pandemic. Because national and state data might obscure what is happening locally, state and local health departments are encouraged to analyze data from their immunization information systems to identify opportunities for increased attention and intervention.

Some of the strategies for helping parents make informed decisions and increasing vaccination coverage include strong, evidence-based provider recommendations, development of targeted messages from credible and trusted sources, and increasing participation in the VFC program (*10*). In addition, the Community Preventive Services Task Force recommends several other interventions to increase vaccination, including standing orders for vaccination, reminders from health care providers, immunization information systems, and vaccination programs in organized child care centers and in Special Supplemental Nutrition Program for Woman, Infants, and Children settings. (The Community Guide | CDC). Additional evaluation of the behavioral and social drivers of vaccination can be helpful for the design of targeted interventions to engage with families about the importance of routine childhood vaccinations and their role in supporting children’s health (*4*,*5*). Interventions such as these can increase vaccination coverage, reverse declines in vaccination coverage associated with the COVID-19 pandemic, and help protect all children from the morbidity and mortality associated with vaccine-preventable diseases.

## Footnotes

* Immunization against respiratory syncytial virus (RSV) using nirsevimab was recommended in August 2023 for infants aged <8 months born during or entering their first RSV season and for infants and children aged 8–19 months who are at increased risk for severe RSV disease and entering their second RSV season. Estimates of nirsevimab coverage are not provided in this report because 1) the first cohort of eligible children was born April 2023–March 2024, but only a small number of children in NIS-Child in 2024 were born during this period, and 2) NIS-Child did not collect data on high-risk conditions in 2024; therefore, assessment of coverage among those who are eligible for nirsevimab during their second RSV season at age 8–19 months was not possible. Another monoclonal antibody, clesrovimab, has been recommended since June 2025, for infants aged <8 months born during or entering their first RSV season who are not protected by maternal vaccination. No preferential recommendation exists between nirsevimab and clesrovimab.

† Vaccination coverage is calculated nationally and for U.S. Department of Health and Human Services regions, states (including the District of Columbia), selected local areas, and the U.S. territories of Guam, Puerto Rico, and the U.S. Virgin Islands (sampled every other year; data were not collected in 2020, 2022, or 2024). Certain local areas that receive federal Section 317 immunization funds are sampled separately and included in the NIS-Child sample every year (i.e., Chicago, Illinois; New York, New York; Philadelphia County, Pennsylvania; Bexar County, Texas; and Houston, Texas). National estimates in this report exclude U.S. territories.

§ Estimates of COVID-19 vaccination coverage are not included in this report. More timely estimates are available online. COVID-19 Vaccination Coverage and Vaccine Confidence Among Children | COVIDVaxView | CDC

¶ VFC eligibility was defined as meeting at least one of the following criteria: 1) American Indian or Alaska Native; 2) insured by Medicaid, Indian Health Service, or uninsured; or 3) ever having received at least one vaccination at an Indian Health Service–operated center, Tribal health center, or urban Indian health care facility. The VFC program provides free vaccines to eligible children for whom cost might otherwise be a barrier to vaccination.

** NIS-Child used a landline-only sampling frame during 1995–2010. During 2011–2017, the survey was conducted using a dual-frame design, with both cellular and landline sampling frames included. In 2018, the NIS-Child returned to a single-frame design, with all interviews conducted by cellular telephone. During 2018–2024, 95%–98% of U.S. adults reported owning a cellular telephone.

†† The Council of American Survey Research Organizations (CASRO) household response rate is calculated as the product of the resolution rate (percentage of the total telephone numbers called that were classified as nonworking, nonresidential, or residential), screening completion rate (percentage of known households that were successfully screened for the presence of age-eligible children), and the interview completion rate (percentage of households with one or more age-eligible children that completed the household survey). CASRO response rates and the proportions of children with household interviews that had adequate provider data for survey years 2015–2023 are available online. NIS-Child: A User's Guide for the 2023 Public Use Data File

§§ Children with at least one vaccination reported by a provider and those who had received no vaccinations were considered to have adequate provider data. “No vaccinations” indicates that the vaccination status is known because the parent or guardian indicated there were no vaccinations and the providers returned no immunization history forms or returned them indicating that no vaccinations had been administered.

¶¶ Before 2020, the first dose of HepA was recommended at age 12–23 months, with the second dose administered 6–18 months after the first. During 2020, the recommendation was revised to 2 doses administered at age 12–23 months, ≥6 months apart, with a catch-up period extending through age 18 years. Because children in this analysis were vaccinated under both recommendations, coverage estimates for both age <24 months and age <35 months are provided.

*** 45 C.F.R. part 46.102(l)(2); 21 C.F.R. part 56; 42 U.S.C. Sect. 241(d); 5 U.S.C. Sect. 552a; 44 U.S.C. Sect. 3501 et seq.

††† The combined 7-vaccine series (4:3:1:3*:3:1:4) includes ≥4 doses of DTaP, ≥3 doses of poliovirus vaccine, ≥1 dose of measles-containing vaccine, ≥3 or ≥4 doses (depending upon product type) of Hib, ≥3 doses of HepB, ≥1 dose of VAR, and ≥4 doses of PCV.

§§§ MSA status was determined on the basis of household reported city and county of residence and was grouped into three categories: MSA principal city, MSA nonprincipal city, and non-MSA. MSAs and principal cities were as defined by the U.S. Census Bureau. Non-MSA areas include urban populations not located within an MSA as well as completely rural areas.
